# Retraction Note: β-Endorphin (an endogenous opioid) inhibits inflammation, oxidative stress and apoptosis via Nrf-2 in asthmatic murine model

**DOI:** 10.1038/s41598-026-68215-0

**Published:** 2026-08-25

**Authors:** Vinita Pandey, Vandana Yadav, Rashmi Singh, Atul Srivastava, Subhashini

**Affiliations:** 1 Department of Zoology, Mahila Mahavidyalya, Banaras Hindu University, Varanasi, 221005 India; 2 Department of Biochemistry, Institute of Medical Sciences, Banaras Hindu University, Varanasi, 221005 India

Retraction of: *Scientific Reports* 10.1038/s41598-023-38366-5, published online 31 July 2023

The Editors have retracted this Article.

After publication, concerns were raised regarding the data presented in the Figures, specifically:

- Fig. 1D TDI and β-END, and Fig. 1E TDI images appear to contain some duplicated features within the images;
- Fig. 8A Multi-sample plot appears to show a combination of the Annexin and PI plots;
- Fig. 8A Control, Vehicle and Endorphin plots appear to share a high number of datapoints.

The Editors therefore no longer have confidence in the presented data.

Vandana Yadav, Rashmi Singh, Atul Srivastava and Subhashini do not agree with this retraction. Vinita Pandey has not responded to any correspondence from the Editors about this retraction.

